# Spontaneous Intracerebral Hemorrhage: Should We Operate?

**DOI:** 10.3389/fneur.2017.00645

**Published:** 2017-12-04

**Authors:** Marc-Alain Babi, Michael L. James

**Affiliations:** ^1^Division of Neurocritical Care, Department of Neurology, Duke University, Durham, NC, United States; ^2^Division of Neurocritical Care, Department of Neurology, University of Florida, Gainesville, FL, United States; ^3^Division of Neurosurgical Anesthesia, Department of Anesthesiology, Duke University, Durham, NC, United States

**Keywords:** intracerebral hemorrhage, brain surgery, cerebral edema, craniotomy, stroke, cerebrovascular disorders, minimally invasive surgery

Spontaneous intracerebral hemorrhage is the second most common type of stroke and is considered the most lethal subtype of stroke. Mortality reaches approximately 50% within the first 3 months and most survivors are left with severe disability (1–3). Despite major advances in the acute emergency neurological life support of patients with ICH, the optimal surgical management of these patients remains controversial (4–6).

In theory, surgical intervention after spontaneous ICH has therapeutic potential; by reducing intracranial pressure, preventing herniation, eliminating the source of hemorrhage, reducing the source of localized mass lesions, and mitigating secondary neuro-inflammatory cascades. Because of this, multiple surgical approaches have been investigated with varying degree of success. Investigated procedures include conventional craniotomy, stereotactic guidance with aspiration and thrombolysis, image-guided stereotactic endoscopic aspiration, and decompressive craniectomy (4–6).

Open craniotomy is the most studied surgical technique after ICH (1, 2, 7). One of the largest meta-analysis of 2,059 patients, concluded that surgery was associated with a reduced risk of death and dependency (OR 0.71; 95% CI 0.61–0.91) compared to medical management alone (8). However, criticisms of this analysis include demonstration of marginal benefit, significant heterogeneity of included studies, and wide variability in the quality of studies. Only two of the selected studies scored positively on all items of methodological quality assessment (7, 9).

To date, two well-powered, randomized controlled trials [Surgical treatment of lobar ICH (STICH) and early surgery versus initial conservative treatment in patients with spontaneous supratentorial lobar intracerebral hematomas (STICH II)] compared surgical evacuation to medical management of ICH (10, 11). The aim of STICH trial was to determine if early hematoma evacuation through open craniotomy decreased death and disability compared to best available medical treatment. Comparing 503 patients with spontaneous supratentorial hemorrhage randomized to early surgery versus 530 patients to initial conservative treatment, intention to treat analyses were blinded. Using the Extended Glasgow Outcome Scale at 6 months, 122 (26%) of patients allocated to the surgical arm had a favorable outcome compared to 118 (24%) in the medical treatment arm (OR 0.89, 95% CI 0.66–1.19). Similarly, mortality at 6 months was 36% in the surgical arm compared to 37% in the medical arm [OR 0.95 (0.73–1.23), *p* = 0.707]. Thus, in this trial, patients with spontaneous supratentorial intracerebral hemorrhage (ICH) had no overall benefit of early hematoma evacuation compared to medical management alone (7).

In 2013, a second trial (STICH II) was conducted to determine if patients in a specific subgroup of spontaneous ICH responded favorably to early surgery (11). The primary outcome of the trial was again the Extended Glasgow Outcome Scale at 6 months after hemorrhage. In limiting enrollment exclusively to patients with lobar hemorrhage, with no evidence of intraventricular extension, as well as excluding comatose patients, the STICH II trial sought to isolate a particular set of patients that seemed to benefit in the subgroup analysis of the initial STICH trial (8). In the medical management arm, 38% of patients had a favorable outcome, compared to 41% in the surgical arm (OR 0.86, 95% CI 0.62–1.20; *p* = 0·367). Similarly, survival during the first 6 months was not significantly different between the medical and surgical groups. Thus, the STICH II trial failed to find sustained benefit of operative management of ICH compared to medical management alone. In both trials, patients were randomly allocated to either surgery or medical therapy using telephone randomization service or internet randomization service, both provided by the Clinical Trial Service Unit at the sponsoring institution. Given the invasive nature of the procedure (surgical group), patients, relatives, and site investigators were aware of which treatments the patient had been allocated to. However, at the coordinating center, only the data manager was aware of the allocation. Interestingly, and in the STITCH II trial, around 50% of the patients were allocated using simple randomization alone. Nevertheless, this did not compromise the study and such randomization was undertaken by the investigators to overcome any imbalance in overall numbers.

However, both trials failed to show an outcome benefit of surgery over medical management alone. Nonetheless, when the data from the STITCH II trials are combined with results from additional prior trials, the results suggest a potential survival-benefit for the surgical group over medical therapy alone, particularly in the subgroup of patients who have a poorer prognosis on presentation, secondary deterioration, and superficial ICH but no intraventricular extension (10–12). Given the heterogeneity in the quality of prior studies, one need to cautiously interpret such data, and further studies are required to conclusively determine which patients should optimally receive surgical therapy. Additionally, criticisms of STICH II included a great number of patients excluded (>3,300) for lack of “consciousness” at randomization. Nevertheless, conscious patients at randomization likely reflect those with less severe ICH, and in turn, a higher likelihood of good outcome, regardless of group assignment. Excluded patients with impaired consciousness due to hematoma expansion or brain herniation may not have allowed equipoise for enrollment since they may have been likely candidates for surgery as a life-saving measure. In addition, cross-over to the surgical arm was significant, and the authors dichotomized entry groups into “good” and “poor” prognosis based on prognostic scores. Despite negative findings, further stratification through subgroup analyses of STICH II may identify a group of patients responsive to open hematoma evacuation. In fact, large meta-analyses that included STICH II data suggest surgical benefit for select subgroups of patients, including those with poorer prognosis at presentation, those with secondary deterioration attributed to hematoma expansion, and those with superficial hematomas without intraventricular extension (5, 6, 9, 12).

As an alternative to open craniotomy for hematoma evacuation, minimally invasive and stereotactic surgical techniques are currently being evaluated. The intraoperative stereotactic computed tomography-guided endoscopic surgery (ICES) study suggested that early computerized tomographic image-guided endoscopic surgery is a safe and effective method in select cases to remove acute intracerebral hematomas (5). Similarly, the minimally invasive surgery plus alteplase^®^ in intracerebral hemorrhage evacuation (MISTIE) trial found catheter-based hematoma removal to be safe and promising (6). However, questions remain regarding the surgical optimization of these minimally invasive techniques, including patient selection and timing of surgery. Efficacy phase trials for both stereotactic endoscopic (ENRICH) and catheter-based (MISTIE III) techniques are currently enrolling. The ENRICH trial is a multicenter, randomized, adaptive clinical trial that aims at comparing standard medical management to early surgical hematoma evacuation (less than 24 h) using minimally invasive parafascicular surgery in the treatment of ICH (13). The MISTIE III trial is a multicenter randomized controlled trial that aims at comparing standard medical management in ICH versus use of minimally invasive surgery (MIS) plus recombinant tissue plasminogen activators (rt-PA) for 3 days (14). Most recently, data from a pilot study presented at the Congress of Neurological Surgeons suggested that the use of the FDA-approved BrainPath^®^ device to perform MIS in select patients may result in improved outcomes, reduced length of stay, reduced operation times, and the ability to perform awake craniotomy (15, 16).

In summary, the efficacy of surgical hematoma evacuation for patients with ICH compared to medical therapy, remains an open debate. Multitudes of studies were performed, of different methodologies, heterogeneity of techniques and patients’ selection. Some subgroups of patients may respond to surgery whereas other may not. However, the exact patients’ characteristics, timing of surgery, surgical techniques, and postoperative care remain unclear. Nonetheless, there is insufficient evidence to recommend for or against a particular surgical protocol at the moment, but based on the current best available data, hematoma evacuation may be life saving in certain patients yet without the long-term improved neurological function.

## Author Contributions

M-AB and MJ report no disclosures. No funding was obtained to prepare or publish this manuscript. M-AB and MJ contributed to the preparation and drafting of this manuscript. All authors have read and approved this manuscript in its final form.

## Conflict of Interest Statement

The authors declare that the research was conducted in the absence of any commercial or financial relationships that could be construed as a potential conflict of interest.

## Abbreviations

ICH, intracerebral hemorrhage; STITCH, surgical treatment for intracerebral hemorrhage; STICH II, surgical trial in lobar intracerebral hemorrhage; ICES, intraoperative stereotactic computed tomography-guided endoscopic surgery; MISTIE, minimally invasive surgery plus alteplase^®^ in intracerebral hemorrhage evacuation; ENRICH, early MiNimally-invasive removal of intracerebral hemorrhage; rt-PA, recombinent tissue plasminogen activator; MIS, minimally invasive surgery; MIPS, minimally invasive parafascicular surgery.
